# Atypical Cavernous Sinus Thrombosis: A Diagnosis Challenge and Dilemma

**DOI:** 10.7759/cureus.3685

**Published:** 2018-12-04

**Authors:** Tong Jong Haw Matthew, Adil Hussein

**Affiliations:** 1 Ophthalmology, Universiti Sains Malaysia, Kota Bahru, MYS; 2 Ophthalmology, Universiti Sains Malaysia, Kota Bharu, MYS

**Keywords:** cavernous sinus thrombosis

## Abstract

Cavernous sinus thrombosis (CST) is an ocular emergency because of its devastating effect and it is prone to cause serious complications. Diagnosis of cavernous sinus thrombosis is a challenging task despite medical advancement. Efforts to promptly diagnose and initiate treatment require a high index of suspicion and a deep understanding of the disease. Unfortunately, patients suffering from CST may not always present with typical symptoms, thus making diagnosis all the more challenging. We would like to describe a 22-year-old man who presented with atypical symptoms and radiological presentations of CST. The patient was admitted and treated in our institution with full recovery demonstrated after the treatment.

## Introduction

Cavernous sinus syndrome is an ocular emergency. Progression is usually fast with devastating effects and can be life threatening. Majority of cavernous sinus thrombosis (CST) arises from trauma and paranasal sinus infections attributing to *Staphylococcus aureus* infection [1]. Due to the anatomical configuration, an intracranial extension is common with a high mortality rate [2]. Early diagnosis of CST and treatment has reduced the mortality rate to less than 20%; however, significant morbidity and long-term complications such as loss of function, hemiparesis and blindness may still occur [1].

## Case presentation

A 22-year-old man presented with a complaint of left-sided non-radiating headache throughout the day for two months, with the pain worsening at night and associated with non-projectile vomiting. The patient managed to achieve some relief with oral analgesia. There were no symptoms of raised intracranial pressure. His symptoms worsened a week before the presentation of gradual painless reduction of his left visual field, particularly on the superior and inferior aspects. He also noticed binocular double vision with significant deviation of his eye. There was no history of recent head trauma, upper respiratory tract infection or sinusitis. He also denied any medical or surgical procedures, allergies and recreational or prescription drug use. He was a smoker of five pack years. 

On examination, his vital signs were stable with no documented fever, and he appeared well, alert and conscious. On further examination of the visual system, his visual acuity (VA) of the right eye was 6/6, while the left eye VA was 6/7.5. There was partial ptosis of the left eye covering the visual axis with gross axial proptosis and mild exotropia of the left eye. There were, however, no signs of dilated conjunctiva vessels, cork-screw vessels or conjunctiva chemosis. Measurement with a Hertel exophthalmometer for the right and left eyes showed 15 mm and 22 mm, respectively, with a discrepancy of 7 mm between both eyes. Examination of the optic nerve function revealed no relative afferent pupillary defect (RAPD) and no defect in the Ishihara pseudoisochromatic plates for both eyes, but a relative 30% reduction in red saturation and light brightness of the left eye compared to the right. Extraocular movement of the left eye was limited for superior, temporal, inferior and nasal gaze. The patient also complained of binocular diplopia in all gazes. Examination of the anterior and posterior segments of both eyes appeared normal. Examinations of all cranial nerves were normal. Other related examinations revealed the absence of sinusitis, dental caries or odontogenic cause, otitis, facial furuncles or any breaks of skin around the face. There were no scalp abscess or any enlarged cervical lymph nodes.

**Figure 1:**
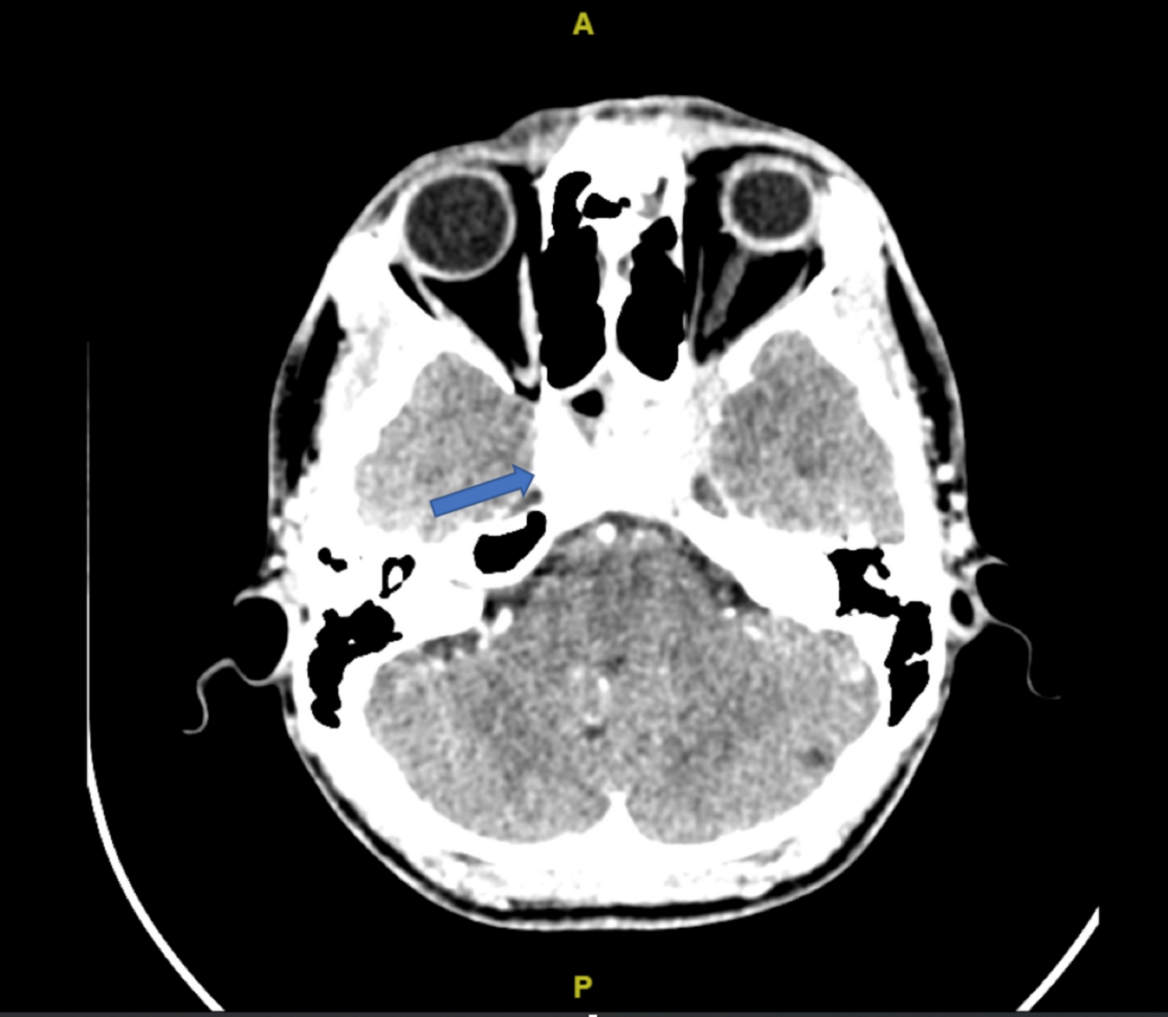
CT scan of the cavernous sinus Arrow pointing towards vascular-enhancing lesions on the right cavernous sinus with filling defects especially at the posterior part. CT: computed tomography

Computed tomography (CT) venogram was done instead of the magnetic resonance imaging (MRI) because of resource constraints, and CT venogram was the best imaging modality that could be offered to the patient at the time. CT venogram (Figure 1) was done, which revealed normal dural venous sinuses with vascular enhancing lesions on the right cavernous sinus with a filling defect, especially at the posterior part. Poor delineation of the cavernous segment of the right internal carotid artery was also observed with the left cavernous sinus concavity preserved. There was no dilatation or tortuosity of the bilateral superior ophthalmic vein with no filling defects. Other findings include mucosal thickening of the sphenoid, ethmoidal and bilateral maxillary sinuses, but the other sinuses and mastoid air cells were well aerated. There were no other significant brain findings. In view of strong clinical signs and radiological features that were strongly suggestive of CST, the patient was admitted urgently and started on intravenous ceftriaxone, intravenous cloxacillin and intravenous metronidazole with the diagnosis of the left cavernous sinus thrombosis in mind. The patient completed the intravenous antibiotics in a week and was discharged well with good vision in both eyes, reducing proptosis, resolved exotropia and binocular diplopia. Review in a week’s time showed marked clinical improvement with the patient achieving total clinical resolution in a month’s time.

## Discussion

CST refers to clotting of the cavernous sinus that may have originated from an infective or aseptic cause. Aseptic causes are typical after surgical procedures or trauma related [1]. Surrounding infections such as pre-septal or orbital cellulitis, sinusitis or otitis can result in CST due to the proximity of these structures and the characteristics of the veins and sinuses that lack valves [2]. CST originating from paranasal sinuses is about 15%, while most cases originate from nasal or mid-facial skin infections [3]. Infections that are commonly isolated are *Staphylococcus aureus* (about 70%) and *Streptococcus* species (about 20%). Other less commonly isolated organisms include *Pneumococcus*, *Haemophilus*, *Pseudomonas*, *Bacteroides*, *Corynebacterium*, and Aspergillus [1,4].

Patients with CST may present early with symptoms of pyrexia and signs of septicemia such as tachycardia, hypotension, rigor and confusion [1]. The most common presentation is a headache (50% to 80%) that is described as unilateral, frontotemporal or retrobulbar, followed by purulent nasal discharge, pharyngeal discharge, inflamed nasal mucosa and tenderness of the sinuses [5]. Signs of meningism such as neck stiffness can be demonstrated in about one-third of the patients [1,6]. Symptoms in the eye include rapid-onset proptosis, ptosis, chemosis, congestion of the conjunctiva, reduced vision and reduced ocular movements [3]. Other significant signs include a rise in intraocular pressure, dilatation of the veins as well as swelling and disc ischemia. Visual impairment has been reported in 7% to 22% of the cases with blindness up to 8% to 15% of the cases [1]. These signs can be attributed to venous congestion secondary thrombosis of the tributaries and drainage with the impairment of ocular movement starting with a lateral gaze. The restriction of eye movement can progress to limiting all gazes if involving cranial nerves located within the cavernous sinus. 

The diagnosis of CST requires a sense of urgency, strong suspicion from clinical findings [3]. Radiography tests such as high-resolution contrast-enhanced CT or MRI as a non-invasive and an efficient diagnostic tool have remarkably assisted clinicians in improving the diagnosis of CST. The direct signs of CST on the contrast-enhanced CT scan include expansion of the cavernous sinus, the convexity of the lateral wall and abnormal filling defects within enhancing CST (septic thrombosis of CST). Indirect signs include concomitant venous obstructions, for example, dilatation of superior ophthalmic vein, exophthalmos, soft tissue edema, and thrombus in veins and sinuses of cavernous sinus tributaries [7]. The advantage of using MRI compared to CT in detecting septic CST is due to its ability of multiplanar sections and details of the blood vessels [3]. Advancements of the radiological investigations and early intervention have significantly improved patient’s recovery and reduced morbidity.

The clinical presentation from the case report describes a patient with atypical features of cavernous sinus thrombosis. Symptoms started with a headache and periorbital pain but devoid of any paranasal sinus infection or any history of head trauma. The examination also revealed a non-inflamed eye, proptosis, variable reduction of VA, negative RAPD and variable changes in optic nerve function test. Urgent CT scan was done for this patient revealing thickened sphenoid, ethmoidal and maxillary sinuses, which may indicate recent upper respiratory tract infection even though the patient did not present with any symptoms. The infection may spread from these sinuses due to the proximity of the structures [3]. Unfortunately, a CT scan of the brain and paranasal sinuses (rather than an MRI) was done due to hospital limitation, especially in the urgent setting. 

Komatsu et al. [3] reported a similar case of cavernous sinus thrombosis caused by a contralateral sphenoid sinusitis attributing the pathology to the proximity of the cavernous sinuses and paranasal sinuses [3]. The anatomical importance of sphenoid sinus is due to its proximity and the anatomical location situated between both cavernous sinuses. Another attributed risk to the anatomical structure of the cavernous sinus, which Imholtz et al, [8] mentioned, was the “valveless” nature of the cavernous sinus that allows a multidirectional flow. Another peculiarity lies in the contrasted CT finding that demonstrates thrombosis on the right side of the cavernous sinus rather than the left despite the patient’s presentation. This could be attributed to the proximity and limited space within the cavernous sinus, causing compression of the nerves on the contralateral wall [3]. We would also postulate that despite preserved concavity of the left cavernous sinus, there might be an area of clotting that may not be visible on CT angiography, giving rise to the signs demonstrated for the left eye.

The treatment of CST should take priority because of the nature of the disease and the severe morbidity and mortality risk that can arise. The risk of visual impairment of the affected eye in 7% to 22% of the patients suffering from CST, with blindness reported in 8% to 15% of the cases [1]. Besides ocular involvement, systemic complications such as meningitis, encephalitis, brain abscess, pituitary infection, epidural and subdural empyema, coma and death may be due to an intracranial extension of infection [2]. In view of the severity with high morbidity and mortality rates of CST, the treatment and procedure should be prioritized and commence immediately. Intravenous antibiotics have greatly increased the survivability while reducing morbidity and mortality of the CST patients. A broad spectrum of antibiotics should be chosen based on the common commensals and source, such as sinusitis, dental abscess or facial cellulitis, such as third-generation cephalosporin, nafcillin and metronidazole. In cases of methicillin-resistant organisms, vancomycin can be a substitute to nafcillin [2]. Treatment should preferably commence after the clinical diagnosis while awaiting any radiological investigation or culture results. Treatment can be later tapered according to the results of the culture and the organisms isolated and their sensitivity. There is no exact duration of the treatment, but treatment is recommended to be at least two weeks or extended beyond clinical resolution to treat any sequestration within the thrombus [1,3]. Surgical treatment and drainage of the paranasal sinuses can be done endoscopically if CT scans demonstrated collections [9]. This is important to remove the source of infection, aerate and restore the normal mucociliary flow of the paranasal sinuses [3]. Other treatments such as the use of heparin and steroids remain controversial [3-5,10].

## Conclusions

The diagnosis of CST remains a challenge despite advancements in radio-imaging technologies. Clinicians need to have a high index of suspicion as clinical features are still the key to an early diagnosis and treatment initiation for patients with CST. When in doubt, the opinion of otolaryngologist should be sought to assist in identifying the paranasal source of infection as delay in treatment would result in devastating risks of morbidity and mortality for the patient.
